# Microbiome for Mars: surveying microbiome connections to healthcare with implications for long-duration human spaceflight, virtual workshop, July 13, 2020

**DOI:** 10.1186/s40168-020-00951-5

**Published:** 2021-01-04

**Authors:** Michael LaPelusa, Dorit Donoviel, Sergio E. Branzini, Paul E. Carlson, Stephanie Culler, Amrita K. Cheema, Rima Kaddurah-Daouk, Denise Kelly, Isabelle de Cremoux, Rob Knight, Rosa Krajmalnik-Brown, Stephen L. Mayo, Sarkis K. Mazmanian, Emeran A. Mayer, Joseph F. Petrosino, Keith Garrison

**Affiliations:** 1 Department of Medicine, Vanderbilt University Medical Center, One Hundred Oaks – North 719 Thompson Lane Suite 20400, Nashville, TN 37204 USA; 2 Department of Pharmacology and Chemical Biology, Center for Space Medicine, Baylor College of Medicine, One Baylor Plaza, Houston, TX 77030 USA; 3 Department of Neurology, Weill Institute for Neurosciences, University of California, San Francisco, CA 94158 USA; 4 Laboratory of Mucosal Pathogens and Cellular Immunology, Division of Bacterial, Parasitic, and Allergenic Products, Office of Vaccines Research and Review, Center for Biologics Evaluation and Research, United States Food and Drug Administration, Silver Spring, MD 20993 USA; 5 Persephone Biosciences Inc, JLABS, 3210 Merryfield Row, San Diego, CA 92121 USA; 6 Department of Oncology, Lombardi Comprehensive Cancer Center, Georgetown University Medical Center, Washington, DC 20007 USA; 7 Department of Psychiatry and Behavioral Sciences, Department of Medicine and the Duke Institute for Brain Sciences, Duke University, Durham, NC 27708 USA; 8 Seventure Partners, 5-7 rue de Monttessuy, 75340 Cedex 07 Paris, France; 9 Departments of Pediatrics, Bioengineering, and Computer Science & Engineering, University of California San Diego, 9500 Gilman Drive, MC 0763, La Jolla, CA 92093-0763 USA; 10 Biodesign Center for Health Through Microbiomes, Arizona State University, Tempe, AZ USA; 11 School of Sustainable Engineering and the Built Environment, Arizona State University, Tempe, AZ USA; 12 Division of Biology and Biological Engineering, California Institute of Technology, 1200 E. California Bl, Pasadena, CA 91125 USA; 13 G. Oppenheimer Family Center for Neurobiology of Stress and Resilience, Ingestive Behavior and Obesity Program, University of California Los Angeles, Los Angeles, CA USA; 14 Vatche and Tamar Manoukian Division of Digestive Diseases, University of California Los Angeles, Los Angeles, CA USA; 15 David Geffen School of Medicine, University of California Los Angeles, Los Angeles, CA USA; 16 Department of Molecular Virology and Microbiology, Alkek Center for Metagenomics and Microbiome Research, Baylor College of Medicine, Houston, Texas USA; 17 Department of Medicine, The University of Texas at Houston Health Sciences Center, 6431 Fannin St, Houston, TX 77030 USA

**Keywords:** Microbiome, Spaceflight, Radiation, Multiple sclerosis, Behavior, Gut-brain axis, Metabolome, Sequencing, Autism spectrum disorder, Fecal microbiota transplants, Live biotherapeutic products

## Abstract

The inaugural “Microbiome for Mars” virtual workshop took place on July 13, 2020. This event assembled leaders in microbiome research and development to discuss their work and how it may relate to long-duration human space travel. The conference focused on surveying current microbiome research, future endeavors, and how this growing field could broadly impact human health and space exploration. This report summarizes each speaker’s presentation in the order presented at the workshop.

## Introduction

An unusual intersection of disciplines took place on July 13, 2020. Microbiome researchers applied their understanding of the human microbiome to challenges facing future long-duration human space flight. The conference was attended virtually by over 500 unique participants from around the globe and was sponsored by the Translational Research Institute for Space Health (TRISH), a NASA-funded consortium led by Baylor College of Medicine in partnership with the California Institute of Technology (Caltech) and the Massachusetts Institute of Technology (MIT). TRISH identifies and funds potentially disruptive scientific discoveries and technological advances that promise to reduce human health risks and performance in long-duration space missions. It does this in part by generating scientific content and opportunities for communities outside of NASA to learn about human deep-space exploration challenges. Virtual conferences engage a broad and diverse audience for exploring how they might apply field-specific knowledge to help safeguard astronauts’ health on the way to Mars.

Drs. Sarkis Mazmanian (Caltech) and Stephen Mayo (Caltech lead for TRISH) developed the conference agenda and recruited the speakers. This report summarizes the presentations and provides context for spaceflight applications. All presentations were videotaped and archived on the TRISH website and can be found here.

Spaceflight imposes many stressors on the human body, including, but not limited to, exposure to space radiation, physical isolation in a closed and unvaried environment, a diet restricted to pre-prepared foods and recycled fluids, decreased musculoskeletal loading secondary to weightlessness, and circadian desynchronization. These stressors have profound impacts on human health, physiology, and biology, as documented over decades of research on astronauts, test subjects participating in analog or simulated missions on Earth, and animal models.

Alterations in different physiological systems occur at different time points during spaceflight missions. Much of the knowledge accumulated regarding these changes is limited to missions shorter than 4 months. As such, minimal evidence exists regarding what changes to human health, physiology, and biology occur during (and after) missions longer than 4 months, and especially for missions longer than 1 year. Notably, as of June 2020, only four individuals have completed spaceflight missions longer than 1 year. Manned missions to Mars could be up to 3 years long. We must understand the exposures and risks to humans associated with long-duration spaceflight so countermeasures can be provided through vehicle design, crewmember schedule, and appropriate resource allocation. It is also essential to study and understand the exposures and risks associated with human spaceflight to improve our understanding of human health and disease generally, which will continue to benefit populations here on Earth.

The human microbiome and different space vehicles’ environmental microbiomes have been interested in the human spaceflight community dating back to the 1960s and 1970s [1, 2]. Previous analyses of crewmembers’ microbiome composition and the microbiome composition of analog mission study participants suggest that several changes to the human microbiome occur with exposure to spaceflight and analog missions. These include changes to alpha and beta diversity, microbial and host gene expression, and shifts between dominant genus [3–7]. Additionally, analyses showed that crewmember microbiomes, particularly of the skin, appear to reflect the space vehicle’s microbiome over time [6, 8]. Moreover, analyses showed that changes to crewmembers’ microbiome composition are reversible upon return to Earth or completion of analog mission (with at least partial reversal occurring on the order of days to weeks) [4, 7]. These findings’ full implications are yet to be demonstrated, and current research focuses on how these changes impact human health and performance.

The explosion of research into the human microbiome over the past two decades has led to associations with obesity, psychiatric and neurological conditions, cancer, allergies, specific autoimmune conditions, and much more. While this field is growing, the current medical utilization of microbiome manipulation is narrow. Perhaps the most well-known example is fecal microbiota transfer (FMT) for recurrent *Clostridium difficile* infection [9]. This same treatment modality has also been used in inflammatory bowel diseases with clinical trials showing promising results [10, 11]. While the success of FMT with *C. difficile* and some inflammatory bowel disease cases is encouraging, the possibility of further therapeutic indications is an area of great intrigue. Other diseases under investigation include microbiome manipulation in irritable bowel syndrome, obesity, autism, nonalcoholic fatty liver disease, and eradication of multi-drug-resistant organisms from colonized individuals [12–15].

Exploration of space has long been a critical driver of scientific and technological advancement and, in particular, has significantly benefited medicine [16]. Our understanding of the microbiome is vital to our continued exploration of space, and this, in turn, may continue to provide new medical advancement for disease management on Earth.

## Workshop goals and objectives

Dr. Stephen Mayo and Dr. Sarkis Mazmanian welcomed the participants and the speakers and explained the goals and objectives of the conference:

1. Survey current understanding and evidence for the effects that the microbiome has on human health.
2. Develop hypotheses about how manipulating the microbiome might be applied as preventative measures or countermeasures to the spaceflight stressors such as weightlessness, isolation, and ionizing radiation.

## A word from the sponsoring organization

Dr. Dorit Donoviel (Baylor College of Medicine), the TRISH director, gave an overview of human spaceflight hazards. She explained why TRISH was interested in the microbiome as an approach to mitigating long-duration space travel stressors. Dr. Donoviel enumerated the many research funding and fellowship opportunities available through TRISH and invited the conference participants to explore funding opportunities or participate as scientific merit reviewers.

## Scientific presentations

The workshop included 12 scientific presentations. Eleven of those presentations are summarized in the following sections. Scientific talk #5, “Microbial pathways to metabolite production” by Dr. Michael Fischbach (Stanford), is omitted.

### Scientific talk #1: perspective on the field and effects of radiation on microbiome

The scientific session was opened by Dr. Rob Knight (University of California, San Diego), who gave an overview of the conference topics. Emphasizing the scale of microbial impact on humans, Dr. Knight briefly touched on how microbes affect food and drug metabolism, how infectious and autoimmune diseases may be related, seasonal microbiome differences, and provided examples of chronic diseases associated with gut microbes [17, 18]. One highlighted topic showed that analyzing microbial rather than human genes could predict over four times more accurately whether someone was obese or lean [19]. Citing studies on skin flora and radiation, antibiotic usage in mice affecting radiation exposure, and the diverse gut flora of voles in Chernobyl, Dr. Knight discussed how radiation and microbiome relate to human spaceflight [20–22]. Another focus was the variance in weight loss and glycemic response concerning diet choice, especially regarding plant consumption [23, 24]. He noted three projects currently underway: American Gut Project, Microsetta Initiative, and Global FoodOmics, all of which study the relationship between what enters and exits the body and what happens between these processes.

Dr. Knight also discussed the use of mass spectrometry in the study of metabolite distribution in mice. Using germ-free (GF) and specific-pathogen-free (SPF) mice, it was shown that the metabolomics data of GF and SPF mice varied drastically throughout the body, and microbiome differences were shown to be responsible for new amino acid conjugations in bile acid [25]. Adapting the mass spectrometry technology used in GF and SPF mice, Dr. Knight showed how the technology could be used to characterize microbiota present in humans [26].

Of particular relevance was the description of Katharoseq, a new utility for identifying microorganisms present in a given area using low-biomass samples. With this technology, it was shown that the Spacecraft Assembly Facility at the National Aeronautics and Space Administration (NASA) Jet Propulsion Laboratory had radiation-resistant organisms present, including *Deinococcus radiodurans* [27]. Along with this, he discussed a study on the microbial communities present on the International Space Station [28]. He detailed how this is important, given the primary objective of reaching Mars and preventing the spread of organisms that originated from Earth. His last discussion topic covered our current understanding of how our microbiome changes in long-term space travel in vivo [6]. Dr. Knight’s concluding remarks emphasized the implications of microbiome research on space exploration and how this may benefit human and environmental microbial research.

### Scientific talk #2: gut microbes in multiple sclerosis: structural, functional, and integrative analysis

Dr. Sergio Baranzini (University of California, San Francisco) spoke about the relationship between the gut microbiome and multiple sclerosis (MS). He described the pathophysiologic link between microbiome dysbiosis, an abundance of pro-inflammatory microbes or lack of anti-inflammatory microbes, and immune dysregulation through pathways that promote T helper 17-cell activity over regulatory T cell activity [29]. This led to a discussion of how previous work demonstrated the role that commensal gut microbes have in triggering immune processes that lead to the development of multiple sclerosis [30]. Dr. Baranzini also discussed how specific bacterial taxa found in higher concentrations in the gut microbiome of MS patients (compared to people without MS) induced pro-inflammatory responses in human peripheral blood T cells, which also likely contributes to the link between the microbiome, immune system, and MS. [31]

Dr. Baranzini then spoke about the International Multiple Sclerosis Microbiome study, an international study utilizing a unique household case-control design to determine how individual patients’ microbiome impacts multiple sclerosis regarding susceptibility, progression, and response to treatment. Preliminary results of this study were shown and included how geography significantly influences a person’s gut microbiome, which can be attributed to many factors, including the relationship between geography and diet.

Lastly, Dr. Baranzini gave an overview of how knowledge network-based multi-model data integration, specifically using SPOKE (Scalable Precision Medicine Oriented Knowledge Engine), combines different data types into one environment and ultimately allows new connections between data to be identified [32]. Several implications for patient care, as well as human spaceflight, were reviewed. Dr. Baranzini outlined how entering gene expression profiles of space-flown mouse liver, spleen, and thymus (and comparing to ground-based controls) in SPOKE could allow users to compare and contrast gene expression profiles seen in tissues stressed by disease not previously thought to be related to spaceflight. Thus, SPOKE can elucidate links between spaceflight-related stress on the human body and common medical conditions at the molecular level that were previously unknown and could lead to targets for disease prevention and therapeutic intervention.

### Scientific talk #3: gut-brain connections to behaviors in mice

Dr. Sarkis Mazmanian (Caltech) began his talk by discussing the origins of his work on the “gut-brain axis” concerning autism spectrum disorder (ASD). He discussed using the maternal immune activation (MIA) mouse model for the study of ASD [33]. This mouse model has been shown to display behaviors similar to those seen in humans with ASD, such as decreased communication, repetitive behaviors, anxiety, and impaired social interaction. As MIA mice were shown to have “leaky gut”, a gastrointestinal disturbance also seen in humans with ASD, mice were given the human commensal *Bacteroides fragilis* to restore gut health. Probiotic treated mice subsequently showed increased communication, decreased repetitive behaviors, and decreased anxiety [34]. Additionally, MIA mice were shown to have increased levels of specific serum metabolites, including 40-fold higher levels of 4-ethylphenylsulfate (4-EPS), which also returned to normal levels after *B. fragilis* treatment [34]. Recent human studies have shown up to 9-fold increases in 4-EPS among some individuals with ASD compared to typically developing controls [35]. Subsequently, mouse studies were devised that compared animals harboring a gut microbiome that produces 4-EPS (4-EPS+) or does not (4-EPS−). Mice exposed to 4-EPS in the gut displayed increased oligodendrocyte precursors (OPC) with decreased mature oligodendrocytes and reduced myelination in their brains compared to mice not treated with 4-EPS. Importantly, 4EPS exposure led to anxiety-like behaviors and certain ASD-like behaviors in mice. Chemical maturation of OPCs to mature oligodendrocytes restored myelination and behavioral changes. These studies show that a specific microbial metabolite, elevated in human ASD, can affect brain activity and function and complex behaviors in mice. While not addressed in the talk, these studies may have implications for behavioral changes associated with spaceflight.

### Scientific talk #4: gut-brain connections to behaviors in humans

Dr. Emeran Mayer (University of California, Los Angeles) opened his talk emphasizing the complexity and bidirectional characteristics of the brain-gut microbiome (BGM) interactions. He emphasized the importance of circular communication within the BGM system, illustrated by the fact that stress-induced alterations in gut function can lead to changes in gut microbial composition and production of microbial metabolites. Some of these neuroactive metabolites signal back to the brain, potentially affecting emotion regulating and central autonomic networks. In turn, this feedback regulation can alter the autonomic nervous system output to the gut and its microbiome.

He further discussed the detrimental effects the Western diet, chronic stress, and other factors can have on the integrity of the gut barrier function leading to a reduced number of mucus stimulating microorganisms, a compromised mucus layer, and increased gut permeability, allowing access of gut microbes to the gut-based immune system (“leaky gut”) [36]. Such low-grade systemic immune activation associated with the Western diet and chronic stress has been implicated in several brain disorders, including depression and neurodegenerative diseases. He emphasized that several large clinical studies and preclinical evidence strongly support a crucial role of a diet high in plant-based components favoring gut microbial diversity in preventing and possibly treating some of these brain disorders. Using the example of interactions between dietary tryptophan, and the generation of different tryptophan metabolites, including serotonin in the brain-gut microbiome network, he spoke about the different windows during development in which gut microbial metabolites may exert lasting effects on the brain, highlighting the temporal associations between gut microbial composition and developmental, metabolic, affective, and neurodegenerative disorders [37]. Using the example of depression and the possible role of the gut microbiome in its pathophysiology, Dr. Mayer briefly discussed a large study of the relationship between depression and host-microbiome, noting how this human study, in addition to many others, can only draw conclusions related to association, not causation [38–42].

Of particular importance was a discussion on neurocognitive studies, particularly in Alzheimer’s and Parkinson’s disease [43–45]. He examined one study in particular, which showed the coexistence of constipation with rapid eye movement sleep abnormalities as a predictor of subsequent development of Parkinson’s disease onset decades later [46]. Dr. Mayer concluded his presentation with a note of caution, stating that it is essential to examine gut microbiome in conjunction with genetic susceptibility and environmental exposures leading to disease, not one factor alone [47]. Not taking into account this complexity may contribute to the difficulties encountered when translating the majority of preclinical studies into clinically relevant and actionable findings.

### Scientific talk #6: microbiome sequencing technologies

Dr. Joseph Petrosino (Baylor College of Medicine) talked about microbiome sequencing technologies. He explained the role of sequencing in translational microbiome research, speaking broadly about the experimental pipeline that most translational microbiome projects follow. This pipeline begins with experimental design and progresses to sample collection, storage, shipping, and processing (to include microbial biomass enrichment, deoxyribonucleic acid (DNA), and ribonucleic acid (RNA) extraction and purification), and further to library preparation and quality control-monitored sequencing. The pipeline culminates with the utilization of bioinformatics tools to assess community structure, bacterial pangenomes, genome assembly, and viral detection in samples. He noted that all of these steps could introduce bias into the experiment, with the potential for bias highest earlier in the pipeline and lessened in steps further along the pipeline.

Dr. Petrosino transitioned into strategies, challenges, and advances in microbiome sequencing technology and analyzed spaceflight’s impact on the microbiome. He highlighted specific kits designed to facilitate sampling and storage for challenging cohorts made by Orasure and Norgen. He also explained how universal microbial primers are used for amplifying single or multiple variable regions of the 16S rRNA (ribosomal RNA) gene [48] and highlighted the 4th variable region (V4), which offers an ideal length and proper representation of many bacterial taxa, particularly those that live in the gut. Other variable regions sometimes provide better resolution for specific niches, such as the use of V1-V3 in oral and upper respiratory sites. He outlined some specific sequencing platforms, such as Illumina, PacBio, Oxford Nanopore, and their unique strengths.

Lastly, Dr. Petrosino reviewed The Environmental Determinants of Diabetes in the Young (TEDDY) study. The study investigates the relationship between genetics and the environment (including the microbiome) in developing type 1 diabetes mellitus (T1DM). He reviewed different published data from TEDDY, including how a temporal developmental pattern in the microbiome of study participants was identified [49] and suggested that similar studies could be undertaken to identify factors that could mature or progress the microbiome during spaceflight. He also posited whether viral communities inhabiting the gut microbiome could serve as a biomarker for immune system function. One study identified that enterovirus B infections might be involved in the development of islet autoimmunity [50], a key mechanism in the pathogenesis of T1DM.

### Scientific talk #7: autism clinical trials

Dr. Rosa Krajmalnik-Brown (Arizona State University) opened her presentation with an account of a study that evaluated vancomycin in ASD [51]. She then noted her prior research, which showed that autistic children had lower microbial diversity and fewer “beneficial” organisms, including *Prevotella* species [52]. Dr. Krajmalnik-Brown discussed a recently completed clinical trial that included children with ASD who were given vancomycin for 2 weeks and microbial transfer therapy using standard human gut microbiota over 10 weeks. After completing therapy, subjects were evaluated over 8 weeks and demonstrated reduced scoring on standardized ASD and gastrointestinal symptom rating scales. Also, the children’s stool analysis showed increased bacterial diversity [53]. A follow-up study evaluated gastrointestinal and autism rating after 2 years post-therapy [54]. There was a continued 59% reduction in gastrointestinal symptoms and gradual improvement in ASD rating. The presentation concluded with the announcement of a new double-blind, placebo-controlled clinical trial currently being conducted to study ASD further and gut microbiome interactions in adults.

### Scientific talk #8: gut bacteria human co-metabolism—implications in neuropsychiatric diseases

Dr. Rima Kaddurah-Daouk (Duke) began her presentation by discussing the metabolome as a “biochemical readout” to detect metabolic dysregulation influenced by genome gut microbiome and exposome as a tool to monitor disease states and to determine the efficacy of therapeutics. She discussed research showing human and gut bacteria co-metabolism and partnership in regulating metabolic processes, new technologies to study this, and transitioned into a summary of global initiatives in Alzheimer’s disease (AD) research [55, 56]. Dr. Kaddurah-Daouk discussed a role of cholesterol and its metabolism in AD pathophysiology. She highlighted human enzymes and genes involved in cholesterol biosynthesis and metabolism and gut bacterial enzymes that regulate its clearance through bile acid production. She then reviewed recent studies conducted by the Alzheimer Disease Metabolomics Consortium, where altered bile acid profiles were noted in the blood of Alzheimer patients [45]. Several noted changes were catalyzed by bacterial enzymes leading to increased secondary cytotoxic bile acids that correlated with cognitive decline, amyloid beta and tau brain pathology, and brain neurodegeneration [57]. No bacteria were detected in AD brains and transcriptomic analysis of 1000 brains showed no active synthetic route for these compounds, suggesting that they were transported from blood to brain [57]. These findings highlight the importance of studies that define the gut-brain axis in the pathogenesis of central nervous system diseases and the relevance of the blood-brain barrier.

The presentation concluded with studies on selective serotonin reuptake inhibitors and their impact on tryptophan-dependent gut microbiome metabolites [58] and relevance to the treatment of depression.

### Scientific talk #9: radiation effects on microbiome/metabolome

Dr. Amrita Cheema (Georgetown University Medical Center) addressed the effects of radiation on the microbiome and metabolome. Dr. Cheema started with an overview of how space-relevant radiation modulates the gut microbiome. She referenced a study in which fecal samples from mice exposed to different doses (0.10, 0.25, and 1.0 Gy) of radiation for different amounts of time were analyzed concerning alpha diversity [59]. Perhaps expectedly, alpha diversity was lower in mice that received any dose of radiation. However, alpha diversity was relatively similar in mice exposed to no radiation compared to 1 Gy at 30 days, with less similarity than the mice exposed to 0.10 and 0.25 Gy at 30 days. Dr. Cheema floated the hypothesis of a “hypersensitivity” response to low doses of radiation. In this hypothesis, lower doses of radiation (in this experiment, 0.10 and 0.25 Gy) do not trigger DNA damage repair mechanisms to the extent seen in higher doses of radiation (in this experiment, 1 Gy).

Dr. Cheema also gave an overview of the metabolome and broadly defined metabolomics as the comparative analysis of endogenous metabolites found in biological samples. She reviewed data from the previously mentioned study, showing how space-relevant radiation can cause metagenome changes that are observable at a broader metabolic level. Specifically, pathways involving central carbon metabolism and nucleotide synthesis, among others, were directly affected. She concluded her talk by pointing out the key findings from this study: exposure to low doses of space-relevant radiation shifts the equilibrium in the gut microbiome towards opportunistic pathogens and causes a functional shift in the metabolome towards fermentative metabolism. This leads to pro-inflammatory states similarly seen in pathologic conditions like metabolic syndrome and accelerated aging phenotypes. Dr. Cheema briefly mentioned unpublished data surrounding sulforaphane, a compound found primarily in cruciferous vegetables being explored as a potential dietary countermeasure against alterations induced by space-relevant radiation.

### Scientific talk #10: investment in microbiome space

Dr. Denise Kelly (Seventure Partners) discussed the recent scientific progress made in the microbiome space in her talk focused on the investor landscape in microbiome science and market predictions. She described how the recent history of advances in sequencing increased quality and robustness of data as a result of better sample collection and analytic methods, an explosion in data platforms, and the shift towards more extensive clinical trials with emphasis on human data from animal models are changing the trajectory of the field.

Dr. Kelly showed how in 2013 there were tens of startups in the microbiome space whereas in 2020 there are currently several hundred [60]. She defined multiple areas where current market opportunities exist, including utilizing microbiota for therapeutic purposes (“bugs as drugs”), identifying mechanisms in which microbiota can improve human health (“drugs as bugs”), and how microbiota can be leveraged to intervene with other modalities, such as phage therapy (“drugging bugs”). She noted that there are currently 2540 registered clinical trials leveraging microbiome science worldwide at the time of her lecture.

Dr. Kelly introduced Seventure, a European venture capital firm led by Isabelle de Cremoux that invests in early-stage startups focusing on life science sectors, digital technologies, healthcare, pharmaceuticals, medical technology, biotechnology, nutrition, and food technology. The company has venture capital financings estimated to be $1.5 billion (including public funding). Dr. Kelly showed figures estimating the human microbiome market size to be $352 million in 2018 with a compound annual growth rate from 2019 to 2024 of 26. She highlighted probiotics as the product category currently dominating the market, but with recent increases in investment in other product categories, including therapeutics and nutrition-based modulation of the human microbiome, this will change.

Dr. Kelly then identified recent examples of partnerships between startup companies and larger pharmaceutical companies, equity raises, and initial public offerings in the microbiome space. For instance, Gilead and Second Genome announced a collaboration with a potential deal size of $1.5 billion. Lastly, Dr. Kelly commented on emerging developments such as the microbiome’s role in modulating the immune response to immune checkpoint inhibitors and integrating tools of big data, omics, and machine learning to infer function and provide predictions regarding small molecule targets and prebiotic therapies.

### Scientific talk #11: microbiome in cancer and COVID-19

Dr. Stephanie Culler (Persephone Biosciences) delivered a presentation on the emerging role of the microbiome in cancer therapy. She characterized the health and economic burden globally and specifically in the USA of noncommunicable diseases such as cardiovascular disease and cancer. She also identified a few particular challenges to developing microbiome-leveraging or microbiome-targeting therapeutics, including the lack of established clinical endpoints for trials and challenges in the manufacturing process (e.g., how an anaerobic growth environment is difficult to sustain on a large scale).

Dr. Culler introduced “Poop for the Cure”, a project by Persephone Biosciences aimed at identifying gut microbiota’s role in how patients with cancer respond to cancer therapies. She outlined how the project is acquiring thousands of stool samples from patients with various types of diseases, although the project’s initial focus is cancer. After obtaining samples, whole-genome sequencing is performed. Current areas of study within the project are determining which patients will respond to which therapy for several types of cancer and understanding if it is possible to convert cancer therapy non-responders to responders by shifting the composition of the microbiome.

The final part of Dr. Culler’s presentation touched on how Persephone Bioscience’s artificial intelligence-based prediction technologies have the potential to assess which patients may develop severe disease upon exposure to severe acute respiratory syndrome coronavirus 2 (SARS-CoV-2). She cited several studies indicating how the microbiome has been shown to play a role in vaccine immune response [61–64].

### Scientific talk #12: regulatory considerations for microbiome-based therapeutics

Dr. Paul Carlson (Food and Drug Administration (FDA) Center for Biologics Evaluation and Research (CBER) Office of Vaccines Research and Review - Division of Bacterial, Parasitic, and Allergenic Products) spoke about regulatory considerations for microbiome-based therapeutics. He first described Investigational New Drug (IND) applications, which is a request for FDA authorization to administer an investigational drug to humans. The FDA’s primary objective in reviewing an IND is, in all phases of the investigation, to assure the safety and rights of subjects, and, in phase 2 and 3, to help ensure the quality of the scientific evaluation. Before the submission of a Biologics License Application, there are generally five stages of product development: discovery and preclinical development, preclinical investigations, and phase 1, phase 2, and phase 3 human studies. The IND application and review covers the three phases of clinical investigation. Dr. Carlson then discussed that the FDA encourages “pre-IND” meetings. Typically, for such meetings, the entity which will take responsibility for and initiate a clinical study under IND (the sponsor) provides the FDA with a briefing package including the rationale for the use of the product, product description and proposed indication, the proposed objectives of planned investigations, an overview of the protocol(s) for a planned study, and specific questions for CBER 30 days before the pre-IND meeting. CBER then assembles a review team that comprises diverse scientific expertise and responds in writing to questions provided in the briefing package. The actual pre-IND meeting can then focus on the discussion of items that require further clarification.

Dr. Carlson then spoke about Chemistry, Manufacturing, and Control (CMC) considerations for INDs regarding FMTs and live biotherapeutic products (LBP). He outlined the challenges of regulation of FMTs and posed several questions about ensuring safety—both extrinsic (regarding screening of donors and testing of stool) and intrinsic (regarding unknown long-term health effects of changes to the gut microbiome). He touched on the challenges of safety and mentioned recent safety alerts on FMT products issued by the FDA to the public, including cases of transmission of multi-drug-resistant organisms, enterotoxigenic *Escherichia coli*, and Shiga toxin-producing *E. coli*. He also cited the lack of evidence for or against screening stool samples for SARS-CoV-2. Dr. Carlson then moved to CMC considerations for LBPs. He defined LBPs as biological products containing live organisms applicable to the prevention, treatment, or cure of a disease or condition of human beings that are not vaccines. Specific CMC considerations he mentioned were strain information, antibiotic resistance profiles, information on cell banking systems, description of the manufacturing process, stability data, viable cell data, and bioburden testing (demonstrating absence of extraneous undesirable bacteria). Dr. Carlson spoke about LBP INDs and for certain investigations of commercially available LBPs the process and information required to request a waiver of the requirement to include CMC information in an IND. Sponsors requesting a waiver should submit information showing that (1) the LBP is lawfully marketed as a conventional food or dietary supplement; (2) the investigation does not involve a route of administration, dose, patient population, or other factor that significantly increases the risk (or decreases the acceptability of risk) associated with the use of the LBP; (3) the investigation is not intended to support a marketing application of the LBP as a drug for human use or a biological product for human use; and (4) the investigation is otherwise conducted in compliance with requirements for INDs. If the waiver is not granted, then the sponsor must include CMC information in the IND application.

Dr. Carlson concluded his talk by providing a comprehensive resource he co-authored with colleagues at the FDA to help sponsors navigate the IND application process [65].

Dr. Carlson’s participation in the workshop and contributions to this manuscript is an informal communication and represent his own best judgment. His comments do not bind or obligate the FDA.

## Conclusion

The TRISH-sponsored Microbiome for Mars virtual workshop held on July 13, 2020, surveyed the microbiome field in areas relevant for long-duration human spaceflight. The workshop covered a broad range of areas, from basic science aspects to regulatory considerations for the development of therapeutics. The information presented by the distinguished panelists through their presentations and responses to questions offered by a global audience lays an essential foundation for TRISH to consider in furtherance of its mission to mitigate health and performance risks associated with human spaceflight.

## Data Availability

Not appliable

## Abbreviations

FMT: Fecal microbiota transplant
GF: Germ-free
SPF: Specific pathogen-free
NASA: National Aeronautics and Space Administration
MS: Multiple sclerosis
SPOKE: Scalable Precision Medicine Knowledge Engine
ASD: Autism spectrum disorder
MIA: Maternal immune activation
4-EPS: 4-Ethylphenylsulfate
OPC: Oligodendrocyte precursors
DNA: Deoxyribonucleic acid
RNA: Ribonucleic acid
rRNA: Ribosomal ribonucleic acid
TEDDY: The Environmental Determinants of Diabetes in the Young
T1DM: Type 1 diabetes mellitus
Gy: Gray
SARS-CoV-2: Severe acute respiratory syndrome coronavirus 2
FDA: Food and Drug Administration
CBER: Center for Biologics Evaluation and Research
IND: Investigational New Drug
CMC: Chemistry, Manufacturing, and Control
LBP: Live biotherapeutic product
